# Cardiac Troponin Release after Exercise in Healthy Young Athletes: A Systematic Review

**DOI:** 10.3390/healthcare11162342

**Published:** 2023-08-19

**Authors:** Enric Conesa-Milian, Rafel Cirer-Sastre, Vicenç Hernández-González, Alejandro Legaz-Arrese, Francisco Corbi, Joaquin Reverter-Masia

**Affiliations:** 1 Department of Education Science, Faculty of Education, Psychology and Social Work, University of Lleida, 25003 Lleida, Spain; vicenc.hernandez@udl.cat (V.H.-G.); joaquim.reverter@udl.cat (J.R.-M.); 2 Consolidated Research Group Human Movement Generalitat de Catalunya, University of Lleida, 25003 Lleida, Spain; rcirer@inefc.es (R.C.-S.); alegaz@unizar.es (A.L.-A.); fcorbi@inefc.es (F.C.); 3 National Institute of Physical Education of Catalonia (INEFC), University of Lleida, 25192 Lleida, Spain; 4 Section of Physical Education and Sports, Faculty of Health and Sports Sciences, University of Zaragoza, 50009 Zaragoza, Spain

**Keywords:** troponin, cTn, child, adolescence, exercise

## Abstract

Cardiac troponin (cTn) is a recognized marker used to assess damage to the heart muscle. Actual research has indicated that the levels of cTn increase after doing exercise in individuals who are in good health, and this is believed to be a result of a normal cellular process rather than a pathological one. The main goal of this study was to investigate the evidence of a postexercise release of cTn in child and adolescent athletes (6–17.9 years old) of different ages, sex, and sports disciplines. The Web of Science, MEDLINE, and Scopus databases were used to conduct the research up to March 2023. Three hundred and twenty-eight records were identified from the databases, however, only twenty-three studies were included in the review after being screened and quality-assessed by two independent authors. The gender, age of the participants, maturational status, and training level of the participants, the timing of sample collection, the exercise modality, and the number of participants with values above the cut-off reference were the data analyzed. Males, older young people, and individual sports seemed to have higher levels of serum cTn after practice exercise. Different methodologies, analyzers, and cut-off reference values make it difficult to compare the data among studies.

## 1. Introduction

Cardiac troponin (cTn) is an accepted indicator for the evaluation of myocardial injury, and high serum concentrations are associated with a worse prognosis in patients with and without known coronary artery disease [1,2]. In addition, the T and I isoforms of cTn (cTnT and cTnI) are highly specific proteins related to myocardial cell damage, and they have become key factors for the diagnosis of acute coronary syndromes and necrosis [3]. In this regard, serums cTnT and cTnI are elevated after irreversible heart muscle damage and peak during the subsequent days [4,5]. However, it has been shown that cTnI might have a slightly higher sensitivity and earlier release in cardiac injuries compared to cTnT. This difference in kinetics may have implications for the early detection of myocardial damage [6].

High-sensitivity assays have replaced the standard ones and are able to measure the lowest limits of detection in 99% of the population with a coefficient of variation <10% and detect cTn concentrations in at least 50% of a healthy population at rest [7]. However, this higher sensitivity also allows for some false positives related to etiologies other than acute myocardial infarction (AMI), such as physical exercise [4,8,9]. The fourth definition in the clinical criteria for myocardial infarction (MI) denotes “the presence of acute myocardial injury detected by abnormal cardiac biomarkers in the setting of evidence of acute myocardial ischemia” [10]. However, the detection of an elevated cTn value above the 99th percentile upper reference limit (URL) is used to diagnose a myocardial injury; this is considered acute if there is a rise and/or fall in the cTn values [10], and the reported 99th percentiles for children are lower than in adults for cTn [11,12,13]. A study examined the performance of high-sensitivity cardiac troponin I (hs-cTnI) and high-sensitivity cardiac troponin T (hs-cTnT) in detecting inconsistent diagnoses of myocardial injury based on the European sex-neutral 99th percentile upper reference limits, which were set at 26 ng/L for hs-cTnI and 14 ng/L for hs-cTnT [14]. It is important to highlight that there is evidence showing significant differences in cTnI values in the pediatric population (under 1 year) compared to children and adolescents. Therefore, specific limits should be used [15].

Recently, the scientific literature has shown elevated values of cTnT during and after acute exercise in apparently healthy people [12], which is suspected to be a consequence of a physiological, non-pathological cellular process [16]. One of the hypotheses explains that changes in the cTn might be related to cardiomyocyte membrane injury [17]. Numerous studies have described the kinetics of serum cTn after physical exercise. Serum concentrations increase immediately after exercise, peak at among 3–6 h after the practice of exercise, and go down during the following hours, returning to the basal levels after 24 h of recovery in almost all participants [13,18,19], contrary to an AMI-related release, where the values increase over time. The mechanisms producing the exercise-induced cTn elevations are unknown; however, there are different possibilities. “Exercise could increase cardiomyocyte membrane permeability by mechanical stress, by the production of oxidative radicals, or by preload-induced increase in stretch-responsive integrins” [16]. Meanwhile, another hypothesis says that myocardial cell necrosis is produced as a physiologic non-pathologic reversible process [20,21].

Nowadays, much research has been performed on the release of troponin after exercise in adults and little has been carried out on children and adolescents, considering children to be those who are older than 1 year and younger than 10, and adolescents to be those who are older than 10 years and younger than 18 [15]. The adult population is more frequently studied due to having a higher prevalence and incidence of cardiovascular disease [12,22]. Moreover, adults are more accessible for recruitment, especially in studies that involve high-intensity and/or long duration exercise interventions requiring repeated blood samplings. Most studies generally concur on the positive correlation between the intensity and duration of sports activities and the extendedness of cTn elevation [12,23,24]. Furthermore, some studies have shown that less trained adults have higher levels of cTn after aerobic exercises [19,25,26]; nevertheless, Lopez et al. proved no differences among basketball players [27], and Legaz et al. even demonstrated that elite rowers had higher values after exercise [23]. However, the current data appears to rule out the theory that training has a protective effect on the heart resulting in lower cTn release.

Children and adolescents have been less studied, leading to a lack of consensus regarding the effect of exercise intensity, duration, and type, or individual history or cardiorespiratory fitness levels (CRF) on troponin kinetics, nor on whether there are differences among age groups and sex in the young. Some research suggests that adolescents have elevated cTnT values after the practice of exercise compared to adults [28,29,30]; however, others show slightly different results, showing adults with higher values [24]. When focusing on the kind of exercise, there is also no clear consensus on the matter [23,31].

Basal and exercise-induced cTn elevations in young athletes are intriguing for several reasons. Firstly, the current URLs for cTn are derived from adult cohorts, and there are no established pediatric population values of reference for cTn at present [32]. Establishing specific reference values for children and adolescents would be highly beneficial for clinical practice [33]. Secondly, the immature heart of children experiences higher myocardial work during exercise compared to adults [34]. As a result, it is plausible that the expected values of cTn following exercise might be higher in children and adolescents than in adults, given this physiological difference.

The past few years have seen systematic reviews conducted on this topic, such as those by Cirer et al. [35], Cantinotti et al. [36], and Papamichail et al. [37]. However, all of them had limitations, such as the difficulty of obtaining the data from all studies or the loss of articles during the search process. Therefore, the purpose of this study was to conduct a systematic review to investigate the evidence on the postexercise release of cTn in child and adolescent athletes of different sexes and sports disciplines. We hypothesized that the release of cTn during exercise should be similar between children and adolescents, regardless of CRF and physical activity.

## 2. Materials and Methods

The systematic review was conducted according to the Preferred Reporting Items for Systematic Reviews and Meta-Analyses (PRISMA) statement [38]. The review protocol was registered at the International Prospective Register of Systematic Reviews (PROSPERO) under the identification number CRD42023376226.

### 2.1. Search Strategy

The literature search was performed by two independent researchers who also reviewed the articles independently to screen for eligibility based on the inclusion and exclusion criteria. Once the articles were selected, Zotero.org was used to refuse those that were duplicated. In case of discrepancies, a third author reviewed the unit for consensus.

Searches were carried out in three different databases—the Web of Science, MEDLINE (PubMed), and Scopus—to identify relevant articles from 15 February 2023 to 10 March 2023. According to the investigation question, three different key terms were used: (A) measurement; (B) population; and (C) intervention. In trying to maximize the specificity, the PubMed filter from Cochrane was used. All searches were restricted to the title or abstract, and the keywords were stated in English. The search strategy used was: (“Troponin” OR Troponin OR “Troponin Complex” OR TnT OR “hs-cTn” OR cTn) AND (“Child” OR Children) OR (“Adolescent” OR Adolescence* OR Teen* OR Youth* OR “Female Adolescent” OR “Male Adolescent”) AND (“Exercise” OR Exercise OR “Physical Activity” OR “Physical Exercise” OR “Acute Exercise” OR “Isometric Exercise” OR “Aerobic Exercise” OR “Exercise Training” OR Sport) AND (((randomized controlled trial OR “controlled clinical trial” OR “randomized” OR “placebo”) OR (“clinical trials as topic”) OR (randomly OR trial)) NOT (animals NOT humans)).

### 2.2. Inclusion and Exclusion Criteria

**Figure 1:**
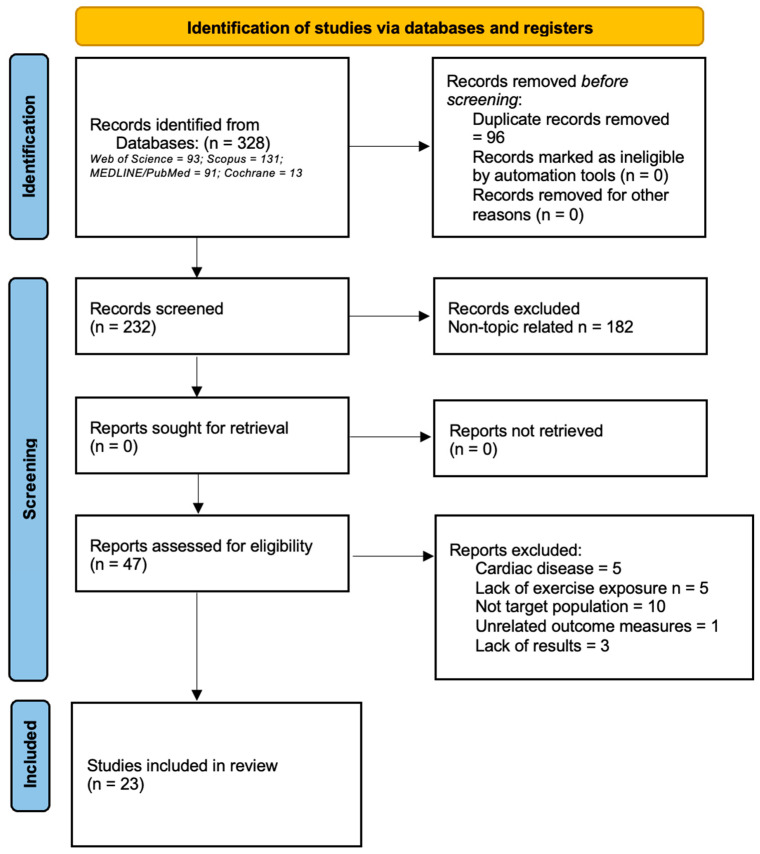
PRISMA Flow Chart of Inclusion and Exclusion Stages.

We selected the studies with a repeated measures design that used an observational or experimental procedure, in which at least one of the intervention groups was under 18. Additionally, all participants engaged in regular physical activity, had no personal history or clinical signs of cardiovascular disease, and exhibited a normal 12-lead electrocardiogram and/or echocardiogram at rest. The interventions examined in these studies involved exposure to physical exercise, including sports events and laboratory tests. Each study was required to assess the cTnT and/or cTnI levels both before and after the exercise exposure. All of the articles identified by the above search strategy were revised to include any quantitative measure of location and variation as the mean (SD), the median with the range, or the median with the interquartile range of the biomarker’s value for a minimum of one time point postintervention. A summary of the search stages can be found in Figure 1.

## 3. Results

### 3.1. Data Extraction

The following data were extracted from the included studies (Table 1, Table 2 and Table 3): the age of the participants (years), maturational status (Tanner Stages from 1 to 5), gender (M/F), training level (years of experience), exercise modality, timing of the sample collection, the levels of serum cTn (ng/L), and the number of participants with values above the URL. The following tables include all of the articles employed to develop the narrative review. If relevant information was unclear or missing, we contacted the researchers to clarify any questions we had.

### 3.2. Quality Assessment and Risk of Publication Bias

To analyze the methodological quality of the studies that met the inclusion criteria, the “Quality Assessment Tool for Before-After (Pre-Post) Studies with No Control Group” created by the National Heart, Lung, and Blood Institute (NHLBI) was used. It contains 12 questions about the different aspects related to the study and the possible answers are yes, no, or cannot determine, or applicable or not reported (Other: CD, NR, NA). This strategy was performed by two independent authors. In the case of any discrepancy, a third solved the problem. It was common among all of the analyzed data that the participants were not random, and many did not have enough participants to provide confidence in the findings; however, the kind of investigation and the characteristics of the population makes it difficult to achieve it.

### 3.3. Variables

#### 3.3.1. Sex

Only 16.52% of the participants were female, out of a grand total of 684 participants, and only 15.20% are females who are not children or adolescents (684-79; n = 605). Furthermore, only 5 out of 23 papers included the female gender in their investigations. Some studies showed that there were no significant differences between sexes [29,43]. In addition, one took the blood sample immediately after the practice and 24 h later, without considering the peak of the cTn serum. The other investigations that included adults and children or adolescents concluded that the males seemed to have higher values post-exercise.

#### 3.3.2. Age

In reference to age, five of the studies compared adults with children or adolescents, while 18 articles only included children or an adolescent population. Three studies had a mixed population, whose standard deviation comprised some older and some younger than 18. In fact, in 65% of the selected studies, all of the participants were under 18. In 22%, we found groups under and over 18, and in the 13%, a group whose standard deviation was over 18 years old was included. Some of them determined the maturational status of each participant; however, this did not happen in all of the studies, making it impossible to compare them.

#### 3.3.3. Training History

All of the articles that have been analyzed in this study included participants with training experience of at least 5 months, either in team sports or individual sports.

#### 3.3.4. Exercise Modality

The exercise modality was different among the studies: 43.5% of them used running as a test to analyze the cTn in the serum, 17.4% adopted swimming as another possibility, and 13% employed soccer as the third most used. The rest of the studies utilized other sports such as table tennis, adventure racing, cycling, basketball, or floorball. A clear predominance of individual exercises existed, which allowed for easy controlling of the time of practice since collective sports, such as football, need a wearable GPS to track the players during the time of practice.

## 4. Discussion

The purpose of this narrative review was to compare how serum cTn kinetics varies in adolescents and children who have been previously exposed to exercise and to analyze which conditions are the determinants. Overall, the review found that the kinetics of serum cTn varied similarly in the subjects, as other authors have already shown in the adult population [11]. However, there were no clear factors or evidence suggesting any specific characteristics that affect the increase in cTn.

Serum concentrations increase immediately after exercise and peak at about 3–6 h after the practice of exercise.

The blood samples in all the studies were taken before and after the physical exercise, and in some articles, they were also taken 2 to 4 h later, the next day, or even two days later. The peak values for cTnT occurred at about 3–4 h post-exercise, and for cTnI, at about 4–6 h post-exercise [21]. This could allow for the estimation of immediate or peak changes, but only a few studies have provided enough measurements to model the serum kinetics of the biomarker. The data also allow for classifying practitioners exceeding the URL. It is important to consider that the subjects were under 18, so how many times could their blood be drawn ethically? There is no specific answer to that question, but it had to be taken into account and ensured that an ethics committee had approved the research.

### 4.1. Sex

Hormones could explain the resting cTnT concentration, for which males had higher values too [13,45,53]. However, there is little evidence regarding females in comparison to males, and there needs to be more research. Cardiac troponin concentrations differ between women and men and are strong predictors of cardiovascular events in women. Using the same thresholds for risk prediction in both sexes would not provide equivalent results [56]. Some authors have studied the menstrual cycle in adult women who do not engage in physical activity, and their results do not show differences between the luteal phase and the follicular phase [57]. However, more research is needed, specifically focusing on female athletes and individuals under 18 years of age.

### 4.2. Age

Some authors said that the levels of hs-cTnT serum depended on the maturational status, explaining that those who were younger had higher values after the exposure to exercise, possibly because of the immature cardiac muscle [23,41,42]. The heart’s internal defense system against harmful molecules, known as anti-oxidative defense, is weaker in young hearts compared to adults. Therefore, young hearts are more vulnerable to oxidative stress, which can significantly increase during exercise [58]. However, recent research shows completely different data, where those who were older had higher values of hs-cTnT serum [13], or they even say that there was no significant difference [53]. If we look at the data of Table 1, Table 2 and Table 3, it seems that the subjects above the URL were higher when they were older. However, only a few studies specified the maturational status, resulting in a small sample of a sum of up to 209, of which 14 were Tanner 1 (2%), 31 were Tanner 2 (5%), 55 were Tanner 3 (9%), 56 were Tanner 4 (9.2%), and 53 were Tanner 5 (8.7%).

### 4.3. Training History

We found three studies that repeated the same effort 2–3 weeks later, and two of them showed lower percentages of subjects with cTn values above the reference limit [50,51]. This could be because their fitness was higher after the rest period and the VTh was not the same. However, the other study proved the opposite, showing a higher percentage of subjects with cTn values above the reference limit in the second effort [52]; this could be due to those who did not participate in the second effort having higher values in the first one. It could be interesting to establish additional groups depending on the years of training, since more experience enables the body to be exposed to multiple situations of physical exercise. Nevertheless, there is not enough evidence to say that the training history was a factor that influenced the cTn serum post-exercise.

### 4.4. Exercise Modality

Looking at Table 1, Table 2 and Table 3, it seems that those studies with more time under tension and controlled as in individual sports had more subjects with values above the URL or the cut-off value, except for one study where the 70% of the pediatric population was above that limit [55]. As other research has shown, the total activity time and intensity controlled by the heart rate are crucial factors in troponin levels. Individual sports allow for more exposure time to a specific workload, thus enabling better control of this key variable [59]. Moreover, we must take into account that combat sports or those that involve higher risks will result in an increase in heart rate. All studies on extreme sports in children have been analyzed, and in this case, one could consider including those that involve participating in a half marathon or even a full marathon. In addition, technical and tactical constraints affect the intensity of the practice in collective sports; this is more chaotic and makes the intensity variable, achieving higher intensities but also periods of lower intensity.

### 4.5. cTn Results above Reference Range

The studies used a third-generation assay to measure the cTn serum, but the diversity of the ability of various assays to detect low levels of troponin is highlighted by the range of the different cut-off values, the lower limit of detection (LLD), the 99th percentile, the 10% coefficient of variation, and the receiver operating characteristics [60,61]. For example, Nie et al. even used the LLD [40,41] and the 10% coefficient of variation [39,42], but there was no consensus, making it difficult to compare the data. The fourth definition of MI [10] was very helpful because everybody was standardized to use only the MI cut-off and stopped using the AMI additionally, with the development of the hs-cTn immunoassays (fourth-generation). The assays had a range of 3–10.000 ng/L, the coefficient of variation at a mean hs-cTnT level of 13.5 ng/L was 5.2%, and the URL for hs-cTnT, defined as the 99th percentile of healthy participants, was 14 ng/L, improving their sensitivity [62]. In case someone wants to analyze the results, they should compare those studies that use the same reference limit and the analyzer.

From a clinical standpoint, it is important for healthcare professionals to recognize that a significant number of individuals may have cardiac troponin (cTn) levels that surpass the upper reference limit (URL) in the initial hours of recovery following both short and long-term exercise. Furthermore, these types of investigations would help determine a specific threshold based on individual characteristics, such as maturation development. Based on the current knowledge, there seems to be no compelling justification for conducting a comprehensive cardiovascular clinical examination in individuals with elevated cTn levels after exercise, unless there are additional clinical indications or symptoms present. Enhancing our understanding of cTn release during exercise holds relevance in enhancing medical decision-making and conducting clinical research among athletes post-exercise.

## 5. Study Limitations

This is a descriptive review which only compiled and analyzed the results derived from a sum of studies independently; however, it is a must to compare the data that all of them gathered to formulate their conclusions. Moreover, it is recommended to include more female participants in the investigations to establish differences between the genders regarding serum cTn and to postulate more robust decisions. Another aspect to think about is working on subjects with different ethnicities; in all these studies, this was not defined, but it could be something to consider. It would also be interesting to assess the hydration level of the participants before and after exercise. Some authors have already shown its relationship in the adult population after engaging in aerobic physical activity [63].

In this work, it was not possible to achieve all of the data necessary to make a comparison among the studies. In this regard, the concentrations of cTn are typically close to 0 and only high in small subgroups, and therefore, the data are commonly asymmetrical. Consequently, cTn data analyses should match their probability distributions using other approaches other than classical parametric tests, which provide uninformative, symmetrical, and too wide confidence intervals, often with unreal cTn serum concentrations below 0. Future research could address this limitation by exploring the individual participant data of the current body of research by unifying and pooling a data analysis.

## 6. Conclusions

Cardiac biomarkers increased in athletes after practicing exercise; however, there were individual responses depending on the variables, such as gender, exercise modality, maturational status, or training level. It is important to consider that duration and intensity are key factors in the release of troponin. It seems that males and older young people had higher levels of cTn after practice exercise, as well as those who practiced individual sports. There is not enough evidence to prove that one’s training history affects their serum cTn kinetics. Moreover, the analyzed studies do not inquire into aspects that other authors may find important in troponin release, such as hydration and right ventricle size, which have been studied in the adult population. Finally, there is no consensus about all of this, as every study uses its own methodology, different cut-off reference values, and even a non-identical analyzer, which makes it difficult to compare the studies.

## Figures and Tables

**Table 1:** Studies and variables analyzed in the Systematic Review in children and adolescents.

Author	Biomarker	Participants Male/Female	Mean Age (Years)	Training Level (Years)	Timing of Sample Collection (Bolded Timepoint Giving Highest Prevalence)	cTn Results above Reference Range	Exercise Modality
Tian, Y., Nie, J., et al. (2006) [39]	cTnT cTnI	10/0	16.2 +/− 0.6	2.4 +/− 1.9	Baseline, 2, **4,** and 24 h after the exercise	cTnT 6/10 (60%) MI cut off (0.03 ng/mL) 4/10 (40%) AMI cut off (0.1 ng/mL) cTnI 6/10 (60%) MI cut off (0.09 ng/L) 1/10 (10%) AMI cut off (0.5 ng/mL)	Half marathon
Nie, J., Tong, T.K., et al. (2008) [40]	cTnT cTnI	10/0	15 +/− 0.7 Tanner: 3.6 +/− 0.5	2.7 +/− 1.2	Baseline, 2, **4**, and 24 h after the exercise	cTnT 4/10 (40%) MI cut off (0.01 ng/mL) 2/10 (20%) AMI cut off (0.05 ng/mL) cTnI 3/10 (30%) MI cut off (0.06 ng/mL) 0/10 (0%) AMI cut off (0.5 ng/mL)	Basketball game (4 × 12′)
Fu, F., Nie, J. & Tong, T.K. (2009) [41]	cTnT	13/0	14.8 +/− 1.6	3.1 +/− 1.5	Baseline, immediately, and **5 h** after the exercise	MI cut off (0.01 ng/mL) AMI cut off (0.05 ng/mL) Group 1: 0/13 Group 2: MI 2/13 (15%); AMI 1/13 (8%) Group 3: MI 8/13 (62%); AMI 3/13 (23%) Group 4: MI 12/13 (92%); AMI 8/13 (62%)	Running (treadmill): Group 1—80% VTh 45′ Group 2—80% VTh 90′ Group 3—100% VTh 45′ Group 4—100% VTh 90′
Fu, F., Nie, J., George, K., et al. (2010) [28]	cTnT	17/0	16.5 +/− 1.6	2.6 +/− 1	Baseline, immediately, and **4 h after the exercise**	13/17 (77%) MI cut off (0.03 ng/mL) 0/17 (0%) AMI cut off (0.05 ng/mL)	Half marathon
Nie, J., Geoge, K., Tong, T.K., Tian, Y. & Shi, Q. (2011) [29]	cTnT	12/0	14.5 +/− 1.5	3.4 +/− 1.5	Baseline, immediately, **POST1+4/PRE2**, POST2, and POST2+4	POST1+4/PRE2: 8/12 (67%) MI cut off (0.01 ng/mL) 3/12 (25%) AMI cut off (0.05 ng/mL)	Running (treadmill): 2 × 45′ VTh. 255′ rest between sets
Nie, J., Tong, T.K., George, K., Fu, F., Lin, H. & Shi, Q. (2011) [42]	cTnT cTnI	12/0	16.2 +/− 0.6	3.2 +/− 1.8	Baseline, 2, **4**, and 24 h after the exercise	cTnT 8/12 (67%) MI cut off (0.03 ng/mL) 8/12 (67%) AMI cut off (0.05 ng/mL) cTnI 11/12 (92%) MI cut off (0.06 ng/mL) 3/12 (25%) AMI cut off (0.5 ng/mL)	Half marathon
Nie, J., Geoge, K., Tong, TK., Gaze, D., Tian, Y., Lin, H. & Shi, Q (2011) [30]	cTnT	53/10	16.4 +/− 1.5	2.4 +/− 1.3	Baseline, **4**, and 24 h after the exercise	57/63 (90%) MI cut off (0.01 ng/mL) 44/63 (70%) AMI cut off (0.05 ng/mL)	Half marathon
Traiperm, N., Gatterer, H., Wile, M. & Burtscher, M. (2012) [43]	cTnT cTnI	19/18	M: 16,7 +/− 0.5 F: 14,7 +/− 1.3	2.9 +/− 1.3 2.0 +/− 1.7	Baseline, **immediately**, and 24 h after the exercise	cTnT 30/37 (81%) MI cut off (0.01 ng/mL) 1/37 (3%) AMI cut off (0.1 ng/mL) cTnI 30/37 (81%) MI cut off (0.1 ng/mL) 2/37 (5%) AMI cut off (0.5 ng/mL)	Marathon
Ma, G., Liu, Y. & Liu, K. (2014) [44]	cTnT cTnI	28/0	7.2 +/− 1.1	6 months to 1 year	Baseline, immediately, **4**, 24, and 48 h after the exercise	cTnT 9/28 (32%) MI cut off (0.03 ng/mL) 5/28 (18%) AMI cut off (0.05 ng/mL) cTnI 6/28 (21%) MI cut off (0.06 ng/mL) 2/28 (7%) AMI cut off (0.5 ng/mL)	Table Tennis (forehand exercises): 6 × 10′; resting 5′ every two sets.
Kong, Z., Nie, J., Lin, H., George, K., Zhao, G., Zhang, H., Tong, TK. & Shi, Q. (2016) [45]	cTnT	19/19	M: 16.1 +/− 1.2 Tanner: F: 3.7 +/− 0.6 Tanner: 4.0 +/− 0.4 15.9 +/− 1.4	M: 2.3 +/− 1.0 F: 2.2 +/− 1.0	Baseline and **4 h after the exercise**	Male 19/19 (100%) MI cut off (0.01 ng/mL) 18/19 (95%) AMI cut off (0.05 ng/mL) Female 18/19 (95%) MI cut off (0.01 ng/mL) 12/19 (63%) AMI cut off (0.05 ng/mL)	Half marathon
Peretti, A., Mauri, L., Masarin, A., Annoni, G., Corato, A., Maloberti, A., Giannattasio, C. & Vignati, G. (2017) [46]	Hs-cTnT	20/0	9.2 +/− 1.7	At least 1 year.	**2.5 h after the exercise**	6/20 (30%) above URL (14 ng/L)	Cycling: “A single, maximal intensity cycling exercise prolonged until muscular exhaustion”
Hosseini, S.M., Azizi, M., Samadi, A., Talebi, N., Hannes, G. & Burtscher, M. (2017) [47]	cTnI	22/0	15.4 +/− 0.4	At least 1 year.	Baseline, immediately, **2,** and 24 h after the exercise cTnI: MI 0	0/22 (0%) above URL (0.035 ng/mL)	Soccer game (90′)
Cirer, R., Legaz, A., Corbi, F., López, I., Puente, J., Hernández, V. & Reverter, J. (2019) [48]	Hs-cTnT	20/0 T2: n = 8 T3: n = 8 T4: n = 4	11.9 +/− 2	5.9 +/− 1.7	Baseline, immediately, and **3 h after the exercise**	4/20 (20%) above URL (14 ng/L)	Soccer: SSG (5 vs. 5) 16′ of effort (4 × 4′) (3′ passive rest between parts)
Cirer, R., Legaz, A., Corbi, F., López, I., George, K. & Reverter, J. (2020) [49]	Hs-cTnT	70/0 T1: n = 14 T2: n = 15 T3: n = 15 T4: n = 13 T5: n = 13	7–18 years	1–11 years	Baseline, immediately, and **3 h after the exercise**	T1: 2/14 (14%) above URL (14 ng/L) T2: 4/15 (27%) above URL (14 ng/L) T3: 6/15 (40%) above URL (14 ng/L) T4: 6/13 (46%) above URL (14 ng/L) T5: 7/13 (54%) above URL (14 ng/L)	Swimming: 6 × 25 m maximal sprints (10” of recovery between efforts)
Birat, A., Bourdier, P., Dodu, A., Grossoeuvre, C., Blazevich, AJ., Amiot, V., Duppont, AC., Nottin, S. & Ratel, S. (2020) [50]	cTnI	12/0	14.4 +/− 0.5	2	Baseline, **immediately**, D+1, and D+2	Race 1 8/12 (80%) MI cut off (0.5 ng/mL) Race 2 6/12 (50%) MI cut off (0.5 ng/mL)	Adventure race: Race 1: 48.2 km Day 1: 5.5 km trail running Day 2: 7.1 km trail running, 27 km mountain biking, 4.4 km kayaking, 4.2 km line skating. **10 days between both races.** Race 2: 66 km Day 1: 7 km trail running Day 2: 14 km trail running, 27 km mountain biking, 18 km kayaking

Abbreviations: cTn, Cardiac Troponin; cTnT, Cardiac Troponin T; cTnI, Cardiac Troponin I; Hs-cTnT, High-sensitivity cardiac troponin T; Hs-cTnI, High-sensitivity cardiac troponin I; AMI, Acute Myocardial Infarction; MI, Myocardial Injury; M, Male; F, Female; C, Children; A, Adult; VTh, Ventilatory Threshold; SSG, Small Side Game; URL, Upper Reference Limit; T1, Tanner 1; T2, Tanner 2; T3, Tanner 3; T4, Tanner 4; T5, Tanner 5.

**Table 2:** Studies and variables analyzed in the Review in children, adolescents, and adults (same groups).

Author	Biomarker	Participants Male/Female	Mean Age (Years)	Training Level (Years)	Timing of Sample Collection (Bolded Timepoint Giving Highest Prevalence)	cTn Results above Reference Range	Exercise Modality
Tian, Y., Nie, J., George, K. & Huang, C. (2014) [51]	Hs-cTnT	10/0	20.4 +/− 5.4	2.4 +/− 0.9	Baseline, immediately, 1, and **3 h after the exercise**	Test 1 10/10 (100%) above URL (14 ng/L) Test 2 9/10 (90%) above URL (14 ng/L)	Treadmill Running 90′: Test 1: 95% VTh **Separated by 3 weeks** Test 2: 95% VTh
Wedin, JO. & Henriksson, AE. (2014) [52]	Hs-cTnT	Game 1: 23/0 Game 2: 16/0	19 (16 to 34 years)	“Elite floorball players”	Baseline, immediately, and **2 h after the exercise**	Game 1 6/23 (26%) above URL (14 ng/L) Game 2 7/16 (43%) above URL (14 ng/L)	Floorball Game 1: 3 periods of 20′ **Separated by 3 weeks** Game 2: 3 periods of 20′
Cirer, R., Jiménez, R., Carranza, LE., George, K., Apple, A., Navarro, R., López, R., Reverter, J., Mayolas, C., Morales, PG & Legaz, A. (2022) [53]	Hs-cTnT Hs-cTnI	24/32 T3: 13 T4: 17 T5: 26	15 (14–22)	2.67 (1.75–5.08)	Baseline, immediately, 1, **3, 6**, 12, and 24 h after the exercise	cTnT 35/62(55%) above URL (14 ng/L) cTnI 28/62 (45%) above URL (11.8 ng/L)	Swimming: 60′ distance trial test.

Abbreviations: Hs-cTnT, High-sensitivity cardiac troponin T; Hs-cTnI, High-sensitivity cardiac troponin I; VTh, Ventilatory Threshold; URL, Upper Reference Limit; T3, Tanner 3; T4, Tanner 4; T5, Tanner 5.

**Table 3:** Studies and variables analyzed in the Review in children, adolescents, and adults (different groups).

Author	Biomarker	Participants Male/Female	Mean Age (Years)	Training Level (Years)	Timing of Sample Collection (Bolded Timepoint Giving Highest Prevalence)	cTn Results above Reference Range	Exercise Modality
Tian, Y., Nie, J., Huang, C. & George, K. (2012) [54]	Hs-cTnT	C: 13/0 A: 13/0	C:14.1 +/− 1.1 T2: n = 8 T3: n = 5 A: 24 +/−3.6	C: 2.7 +/− 1.3 A: 2.5 +/− 1.1	Baseline, immediately, 1, 2, **3**, 4, 5, 6, and 24 h after the exercise	Adults 11/13 (85%) above URL (14 ng/L) Children: Tanner 2 8/13 (62%) above URL (14 ng/L) Children: Tanner 3 4/13 (31%) above URL (14 ng/L)	Running (treadmill): 90-min 95% VTh.
López, I., Legaz, A., George, K., Serveto, O., González, J.M., Reverter, J. & Munguía, D. (2016) [27]	cTnI	PBA: 12/0 ABA: 12/0 JBA: 11/0	27.3 +/− 4.1 29.6 +/− 2.9 16.6 +/− 0.9	17.0 +/− 5.0 13.0 +/− 5.0 8.0 +/− 4.0	Baseline, immediately, 1, 3, **6**, 12, and 24 h after the exercise	PBA 3/12 (25%) above URL (0.04 ng/mL) ABA 0/12 (0%) above URL (0.04 ng/mL) JBA 6/11 (55%) above URL (0.04 ng/mL)	Basketball: 32′ of the 40′. “Every team made a change every 4 min of actual game time”.
Legaz, A., Carranza, L.E., Navarra, R., Valadez, A., Mayolas, C., Munguía, D., Reverter, J. & George, K. (2017) [13]	Hs-cTnT	T3: 4/10 T4: 11/11 T5: 10/4 A: 7/9	14.8 +/− 1.8 15.1 +/− 1.3 16.4 +/− 1.6 31.1 +/− 7.9	2.3 +/− 1.6 2.7 +/− 2.0 4.8 +/− 3.6 7.1 +/− 6.4	Baseline, immediately, 1, **3**, 6, and 12 h after the exercise	T3 6/14 (43%) above URL (14 ng/L) T4 17/22 (77%) above URL (14 ng/L) T5 10/14 (71%) above URL (14 ng/L) Adults 8/16 (50%) above URL (14 ng/L)	Swimming: 5′ Warm up (<60% Hrmax) followed by a 60 min “all out” test.
Cirer, R., Legaz, A., Corbi, F., López, I., Puente, J., Hernández, V. & Reverter, J. (2020) [55]	Hs-cTnT	C: 24/0 A: 12/0	10.7 +/− 1.6 37.5 +/− 12.7	4.6 +/− 1.7 23.6 +/− 14.5	Baseline, immediately, and **3 h after the exercise**	Children 17/24 (71%) above URL (14 ng/L) Adult 8/24 (33%) above URL (14 ng/L)	Soccer: SSG (7 vs. 7) 60′. 4 × 15′ (2′-10′-2′ rest between quarts)
Cirer, R., Corbi, F., López, I., Carranza, LE. & Reverter, J. (2021) [24]	Hs-cTnT	C: 18/0 A: 14/0	14.0 +/− 3 35.0 +/− 9	7.0 +/− 2 6.0 +/− 2	Baseline, immediately, and **3 h after the exercise**	Children 6/18 (33%) above URL (14 ng/L) Adult 8/14 (57%) above URL (14 ng/L)	Swimming: 45′ distance trial test.

Abbreviations: cTnI, Cardiac Troponin I; Hs-cTnT, High-sensitivity cardiac troponin T; Hs-cTnI, High-sensitivity cardiac troponin I; M, Male; F, Female; C, Children; A, Adult; VTh, Ventilatory Threshold; PBA, Adult Elite; ABA, Adult Amateur; JBA, Junior Elite; SSG, Small Side Game; URL, Upper Reference Limit; Hrmax, Maximum Heart Rate; T1, Tanner 1; T2, Tanner 2; T3, Tanner 3; T4, Tanner 4; T5, Tanner 5.

## Data Availability

Coming soon, we will have the data on the project’s webpage, which is currently under construction.
